# Protective effects of gallic acid on cardiac electrophysiology and arrhythmias during reperfusion in diabetes

**DOI:** 10.22038/ijbms.2019.27563.6726

**Published:** 2019-05

**Authors:** Fatemeh Ramezani-Aliakbari, Mohammad Badavi, Mahin Dianat, Seyed Ali Mard, Akram Ahangarpour

**Affiliations:** 1 Department of Physiology, School of Medicine, Ahvaz Jundishapur University of Medical Sciences, Ahvaz Iran; 2 Ahvaz Physiology Research Center, Ahvaz Jundishapur University of Medical Sciences, Ahvaz Iran; 3 Atherosclerosis Research Center, Ahvaz Jundishapur University of Medical Sciences, Ahvaz Iran; 4 Diabetes Research Center, Ahvaz Jundishapur University of Medical Sciences, Ahvaz, Iran

**Keywords:** Cardiac arrhythmia, Diabetes mellitus, Gallic acid, Reperfusion, Rats

## Abstract

**Objective(s):**

Gallic acid (GA), a potent anti-oxidant, plays an important role in reducing diabetic induced cardiac disorders. Therefore, the present investigation was purposed to determine the beneficial effect of GA in cardiac arrhythmias during reperfusion in diabetes induced by alloxan.

**Materials and Methods:**

Male Sprague-Dawley rats (200–250 g) were randomly divided into three groups (eight in each group): control (C), diabetic (D), and diabetic treated with GA (D+G) groups. GA was administered by gavage (25 mg/kg, daily) for eight weeks. Diabetes was induced by a single intraperitoneal injection of alloxan (120 mg/kg). Ischemia-reperfusion (IR) injury was performed by ischemia and then reperfusion (30 and 120 min, respectively). The score and magnitude of arrhythmias, creatine kinase (CK-MB), and lactate dehydrogenase (LDH) of the heart, electrocardiographic, and hemodynamic parameters were measured. One-way ANOVA followed by *LSD* tests were used for the differences between groups. The percentage of incidence was also evaluated by Fisher’s exact test.

**Results:**

The duration (*P*<0.05), onset (*P*<0.01), score and incidence of arrhythmia, QT interval (*P*<0.001), LDH, and CK-MB (*P*<0.05) were significantly elevated and the contractility of the heart (±dp/dt, *P*<0.01), LVSP, QRS complex voltage (*P*<0.05), and heart rate (*P*<0.01) were significantly reduced in the diabetic animals compared with the control rats. However, administration with GA significantly improved these alterations in the diabetic group compared with the diabetic animals.

**Conclusion:**

This study indicated the beneficial effects of GA on cardiac electrophysiology and arrhythmias during reperfusion in diabetes.

## Introduction

Ischemic heart disorder is one of the chief factors of mortality and morbidity worldwide. Reperfusion following a period of ischemia may lead to the alteration of electrical, ionic, metabolic, and ventricular arrhythmias in the heart subjected to ischemia/reperfusion (IR) injury (1-3). Ventricular arrhythmia is one of the lethal consequences of myocardial IR injury (4). 

Diabetes increases the risk of ischemic heart disease and susceptibility to myocardial IR injury (5). This injury is mainly characterized by increasing reactive oxygen species (ROS) in cells. The ROS overproduction leads to oxidation of proteins, lipids, and DNA and oxidative stress. Oxidative stress is involved in cell dysfunction, inflammatory responses, mitochondrial damage, and cell apoptosis (6). Decreased IR injury by treatment with anti-oxidant agents including vitamins C and E and glutathione have been reported (7, 8). Anti-oxidants and free radical scavengers play important roles in protection against myocardial IR injury and reduction of ventricular arrhythmias (9). After IR injury, the susceptibility to oxygen free radicals in the cardiac tissue is increased, resulting from the significant damage to the endogenous anti-oxidant system (10). 

Gallic acid (GA), an effective polyphenol, has anti-oxidant, anti-cancer, anti-mutagenic, anti-diabetic and anti-inflammatory effects and decreases the heart, liver, and kidney injuries (11). GA is found in *red wines*, *gallnuts*, *grapes*, *green tea,* and *barberry* (11, 12). In addition, reduced cardiotoxicity induced by isoproterenol and myocardial dysfunction induced by diabetes with GA treatment have been demonstrated in Wistar rats (13, 14). 

Diabetic cardiomyopathy is associated with cardiovascular diseases and heart failure in diabetic patients (15). The pathological QT prolongation has been indicated to be the main risk factor for mortality and cardiac arrhythmias in diabetes (16). On the other hand, improved QT interval prolongation in the heart by treatment with anti-oxidant agents through reducing ROS and ionic pump dysfunctions under high glucose conditions has been indicated (17). Despite evidence for the beneficial effect of GA on the cardiovascular system, the underlying role of GA in diabetes/reperfusion-related arrhythmias and cardiac electrophysiology are unknown. Therefore, this study was purposed to determine the beneficial effects of GA on cardiac electrophysiology and arrhythmias during reperfusion in diabetes.

## Materials and Methods

***Drugs***

Gallic acid, heparin, and alloxan were bought from the Sigma Company. Magnesium sulfate, potassium chloride, potassium hydrogen phosphate, D-glucose, sodium hydrogen carbonate, calcium chloride, and sodium chloride were purchased from the Merck Company. Xylazine and ketamine were obtained from Alfasan (Woderen- Holland).

***Experimental animals***

The male Sprague-Dawley rats (200–250 g) were bought from the animal house of Jundishapur University of Medical Sciences. The animals were kept in cages at standard conditions (12/12 hr) light/dark cycle, 20–25 °C) in an animal room in the physiology research center and they received water and chow pellets. The present study was confirmed by the Ethics Committee for Research on Animals in Ahvaz Jundishapur University of Medical Sciences (ethics code: APRC-94-25). The rats were randomly allotted to three groups (eight in each group): a control group (C), a diabetic group (D), and a diabetic administered with GA group (D+G). GA (25 mg/kg/day) was administered by gavage for eight weeks. Diabetes was induced using alloxan at 120 mg/kg via single intraperitoneal injection. Four days after alloxan administration, animals indicating fasting blood glucose ≥250 mg/dL were regarded as diabetic rats (16). GA was administered once daily for eight weeks. 

***Experimental protocol***

The animals were anesthetized via IP administration of xylazine, ketamine HCl, and heparin (5 and 50 mg/kg as well as 1000 u/kg, respectively). The animals were ventilated with a rodent ventilator (model: 7025, UGO BASILE) after cannulation of the trachea. The cannula was put in the aorta through a lesion and fixed with the aid of a suture. The isolated hearts were quickly transferred to a Langendorff system and perfused with Krebs-Henseleit solution (constant flow, 8 ml/min, 37 °C, pH = 7.4 and 95% O_2_-5% CO_2_). A balloon (latex) was entered in the left ventricle for left ventricular pressure (LVP) assessment via a Power Lab system and pressure transducer (AD Instruments, Australia). Left ventricle end diastolic pressure (LVEDP) by volume of the balloon was modulated nearly 5–10 mmHg. The minimum and maximum rate of left ventricular pressure (±dp/dt) and LVSP were assessed. All isolated hearts were exposed to ischemia (30 min) after stabilization, which was approved via ST elevation on the electrocardiogram, followed by 60 min of reperfusion.

***Electrocardiography and arrhythmias***

**Figure 1:**
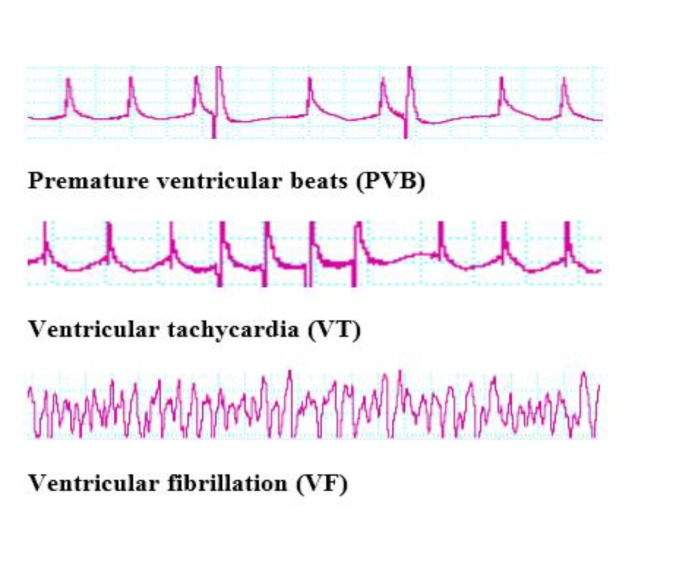
Kinds of arrhythmia

The ECG (Lead II) was systematically controlled via a Power Lab system and pressure transducer (AD Instruments, Australia) during the first 30 min of the reperfusion period. Before ischemia, QRS complex, heart rate, and QT interval in all animals were recorded. Classification of arrhythmias was performed by the Lambeth convention guideline. As shown in Figure 1, an extended QRS complex with ventricular origin was regarded as premature ventricular beat (PVB). Three or more successive PVBs were represented as ventricular tachycardia (VT), and ventricular fibrillation (VF) was started by the incessant lack of QRS (9, 17). Arrhythmia severity was determined by evaluation of arrhythmia score based on the following standards: score 0= PVB< 50 beats; 1=PVB between 50 and 499 beats; 2=PVB > 500 beats or one section of returnable VT or VF; 3=more than one section of returnable VT or VF (<1 min of total combined duration time); 4=1–2 min of total combined duration period VT or VF; 5= more than 2 min of total combined duration period VT or VF (17).

**Figure 2:**
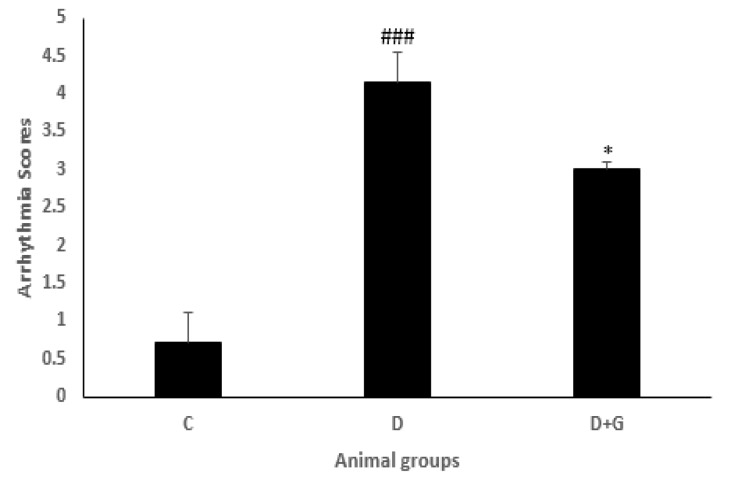
Arrhythmia scores (mean±SEM, n=eight) in control (C), diabetic (D), and diabetic administered with gallic acid (25 mg/kg, D+G). ### *P*<0.001 in comparison with control rats. * *P*<0.05 in comparison with untreated diabetic rats, one-way ANOVA followed by *LSD* test

**Figure 3:**
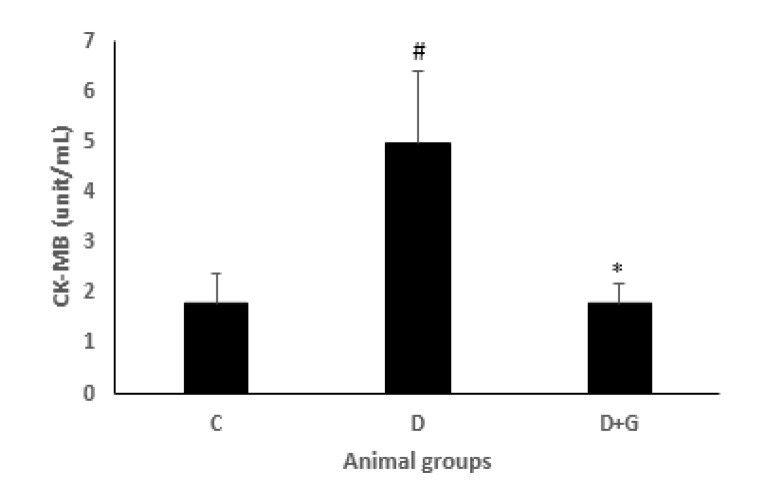
CK-MB level (mean±SEM, n=eight) in control (C), diabetic (D), and diabetic administered with gallic acid (25 mg/kg, D+G). # *P*<0.05 in comparison with control rats. * *P*<0.05 in comparison with untreated diabetic rats, one-way ANOVA followed by *LSD* test

**Figure 4:**
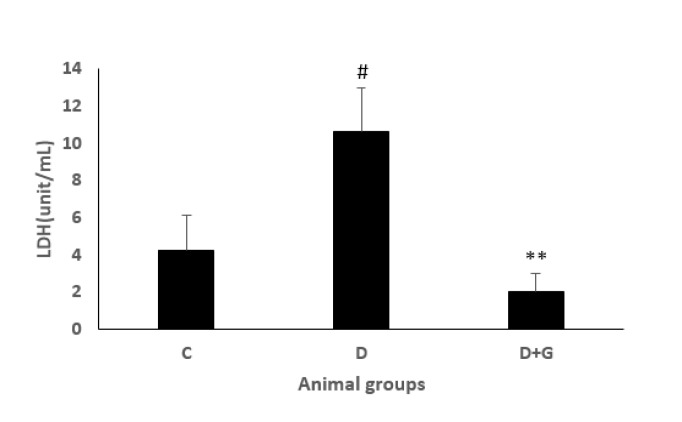
LDH level (mean±SEM, n=eight) in control (C), diabetic (D), and diabetic administered with gallic acid (25 mg/kg, D+G). # *P*<0.05 in comparison with control rats. **P<0.01 in comparison with untreated diabetic rats, one-way ANOVA followed by *LSD* test

**Figure 5:**
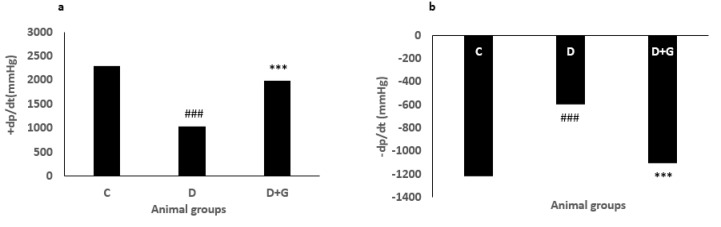
+dp/dt (a) and -dp/dt (b), (Mean±SEM, n=eight) in control (C), diabetic (D), and diabetic administered with gallic acid (25 mg/kg, D+G). One-way ANOVA followed by *LSD* test. ### *P*<0.001 in comparison with control rats, *** *P*<0.001 in comparison with untreated diabetic rats

**Figure 6:**
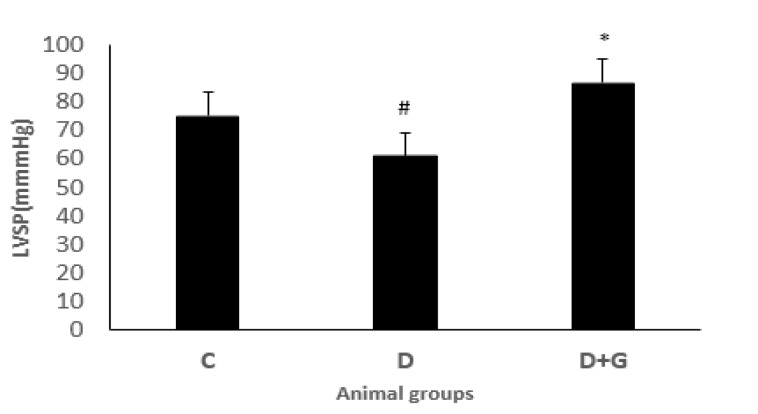
Left ventricular systolic pressure (LVSP, Mean±SEM, n= eight) in control (C), diabetic (D), and diabetic administered with gallic acid (25 mg/kg, D+G). One-way ANOVA followed by *LSD* test. # *P*<0.05 in comparison with control rats, * *P*<0.05 in comparison with untreated diabetic rats

**Figure 7:**
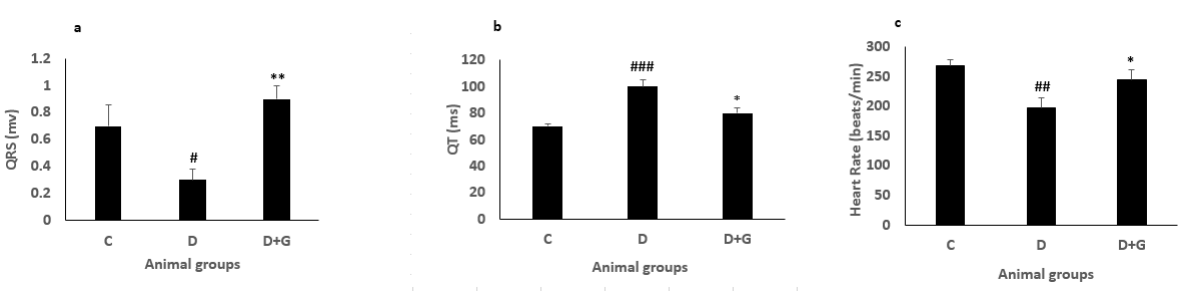
QRS complex (a), QT (b), heart rate (c), (Mean±SEM, n=eight) in control (C), diabetic (D), and diabetic administered with gallic acid (25 mg/kg, D+G). One-way ANOVA followed by *LSD* test. # *P*<0.05, ## *P*<0.01, ### *P*<0.001 in comparison with control rats, * *P*<0.05, ** *P*<0.01 in comparison with untreated diabetic rats

**Table 1:** Gallic acid administration effects on the arrhythmia magnitude in diabetic animals

Groups	C	D	D+G
**Onset of arrhythmia (s)**	**30.37±4.42**	**14.88±2.22** ##	**25.85±3** *
**VT duration (s)**	**2.5±0.5**	**10.44±1.6** #	**4.14±0.6** **
**VF duration (s)**	**0**	**36.1±5.2** ###	**2±0.2** **
**VT (%)**	**28**	**100** #	**100**
**VF (%)**	**0**	**71** #	**12** *

# *P*<0.05,

## *P*<0.01, and

### *P*<0.001 in comparison with control rats,

* *P*<0.05,

** *P*<0.01 in comparison with untreated diabetic rats, (Mean ± SEM, n = eight). One-way ANOVA, followed by *LSD* test and Fisher's exact test). Ventricular fibrillation (VF), Ventricular tachycardia (VT), control (C), diabetic (D) and diabetic administered with gallic acid (25 mg/kg, D+G).

***Cardiac marker enzymes***

Cardiac injury was measured via creatine kinase (CK-MB) and lactate dehydrogenase (LDH) in the heart. At 20 min after reperfusion, perfusate was collected from an isolated heart. The CK-MB and LDH levels were assessed by appropriate kits (Pars Azmoon, Tehran, Iran).

***Statistical analysis***

The data were computed using the SPSS statistical program. One-way ANOVA followed by least significant difference (*LSD)* *post hoc* test were used for the differences between groups. The percentage of incidence was also evaluated with Fisher’s exact test. *P*<0.05 was considered statistically significant.

## Results

***GA effects on arrhythmias***

The onset of arrhythmias significantly occurred earlier in the diabetic group compared with the control rats (*P*<0.01). However, treatment with GA significantly delayed the beginning of arrhythmias in the diabetic animals compared with the untreated diabetic group (*P*<0.05). Incidence percentage and duration of arrhythmias induced by reperfusion were significantly increased in the diabetic rats compared with the control animals (*P*<0.001, *P*<0.05, respectively). In addition, GA administration in the diabetic rats for 8 weeks significantly reduced VT and VF durations (*P*<0.01) and incidence percentage of VF compared with the diabetic group (*P*<0.05) without any changes in VT incidence percentage (Table 1). The arrhythmia score was significantly higher in the diabetic animals compared with the control rats (4.14±0.4 vs. 0.71±0.4, *P*<0.001). Nevertheless, the animals administered with GA presented a lower score of arrhythmia than the diabetic animals not treated with GA (3±0.1 vs. 4.14±0.4, *P*<0.05, Figure 2).

***Effects of GA on CK-MB and*** ***LDH levels ***

The levels of LDH and CKMB in the heart were significantly increased during reperfusion in the diabetic animals compared with the control rats (5±1.4, 10.6±2.4 vs. 1.8±0.58, 4.2±1.9, respectively, *P*<0.05). However, GA administration in the diabetic group significantly decreased LDH and CK-MB compared with the diabetic animals not treated with GA (1.8±0.37, 2±1 vs. 5±1.4, 10.6±2.4, *P*<0.01, *P*<0.05, respectively, Figures 3 and 4). 

***Hemodynamic parameters***

The ± dp/dt and LVSP levels were significantly reduced in the diabetic group compared with the control rats. However, administration with GA significantly improved contractility of the heart and enhanced ± dp/dt and LVSP in the diabetic animals compared with the diabetic animals not treated with GA (Figures 5 a, b, Figure 6).

***Electrocardiographic*** ***parameters***

The ECG record was found normal in the control rats. The QRS complex voltage of the diabetic animals was significantly decreased in comparison with the control animals (0.3 ± 0.08 vs. 0.7 ± 0.16, *P*<0.05). The QRS complex voltage significantly increased by administration with GA compared with the untreated diabetic rats (0.9±0.1 vs. 0.3 ± 0.08, *P*<0.01, Figure 7a). QT interval of diabetic animals indicated a significant increase compared with the control rats (100±5 vs. 70±2, *P*<0.001). QT interval indicated a significant reduction in the GA treated group compared with the diabetic animals (80±4 vs. 100±5, *P*<0.05, Figure 7b). The heart rate of diabetic rats exhibited a significant reduction compared with the control rats (198±15.5 vs. 268±9.8, *P*<0.01). The heart rate showed a significant elevation by GA administration in the diabetic group compared with the diabetic animals not treated with GA (244±17.5 vs. 198±15.5, *P*<0.05, Figure 7c).

## Discussion

Ventricular arrhythmias are important disorders during myocardial IR, which are associated with thrombolysis, angioplasty, coronary spasm, and cardiac surgery under ischemic conditions (18). The present study indicated that cardiac IR led to ventricular arrhythmias, including PVB, VT, and VF in diabetes. Nevertheless, administration with GA for eight weeks resulted in a reduction in the incidence of arrhythmia induced by reperfusion. A reduction in intracellular pH and acidosis induced by anaerobic glycolysis results in electrophysiological alterations in cell membranes. On the other hand, acidosis and the increased proton generation elevate intracellular Na+ by Na+-H+ exchanger during ischemia in the heart. Elevated intracellular Na+ leads to increased intracellular Ca^2+^ level during reperfusion and reperfusion arrhythmias (19, 20). Impaired cardiac rhythm is an important outcome of the cardiac IR in which VF progresses into a fatal arrhythmia. [Ca^2+^]_i_ disturbance is associated with cardiovascular disorders, particularly arrhythmias. In addition, the pathophysiologic mechanism is involved in the development of VT and VF including production of free oxygen radicals and calcium overload in the early stages of reperfusion (21). There is a sudden increase in intracellular Ca^2+^ during myocardial reperfusion that stimulates the mechanism of Ca^2+^ hemostasis in the heart and leads to an increase in intracellular and mitochondria Ca^2+^ results in the death of cardiac cells through increasing cardiomyocyte contraction. A decreased intracellular Ca^2+^ level by sarcolemma Ca^2+^ ion channel antagonists reduces infarct size in the heart. In addition, adenosine triphosphates (ATP) depletion exists in the cardiac myocytes during ischemia/reperfusion; therefore, the Na+-K+ ATPase activity decreases, which results in elevated intracellular Na+ amount and Na+- Ca^2+^ exchanger activity, which increases Ca^2+^ entry and intracellular Ca^2+^ level (22-24). Moreover, there is a metabolite release during reperfusion, which may play a central role in intracellular Ca^2+^ levels and ion channel function. In this study, we demonstrated that GA improved the incidence of arrhythmias induced by IR (25, 26). Inhibited Ca^2+^ influx of L-type Ca^2+ ^channels in isolated thoracic aorta by GA treatment has been reported in rats (27).

Cell membrane damage results in increased membrane permeability and the leakage of CK-MB, CPK, and LDH (28). LDH and CK-MB are important biomarkers for cardiac injury and increased levels of these markers were observed in the present study during reperfusion (29). However, treatment with GA for eight weeks decreased LDH and CK-MB in the coronary effluent. Increased enzymic and non-enzymic anti-oxidants and improved cardiotoxic and nephrotoxic effects induced by cyclophosphamide with GA administration via anti-inflammatory, anti-oxidative, and free radical scavenging effects have been demonstrated (30). Thus, GA through the anti-oxidative effect can partly play the main role in decreasing reperfusion-induced arrhythmias in the diabetic rats. 

Previous studies have reported that connexin 43 phosphorylation was involved in the junction of cell to cell via cardiac gap junctions and reduced phosphorylation of connexin 43 can increase arrhythmias (31, 32). In addition, reduced cellular communication via connexin 43 phosphorylation in the epithelial cells of the liver with GA treatment has been indicated in rats (33). Therefore, it is possible that connexin 43 phosphorylation plays an essential role in the induction of cardiac arrhythmias during IR injury. Moreover, the anti-arrhythmic effect of NO by regulating calcium homeostasis and increasing NO level, eNOS expression, SOD and GPx activities by propyl gallate, an alkyl ester of GA have been revealed (34, 35). 

Diabetic cardiomyopathy is associated with diastolic and systolic dysfunctions and impaired intracellular calcium, sodium, and potassium (36). ±dp/dt are cardiac diastolic and systolic indexes and used for the evaluation of contraction and relaxation (37). Reduced ±dp/dt and LVSP has been indicated in diabetic rats, which is consistent with this study (38). The QT interval is the main marker of cardiac infarction and death in diabetic patients (39). In our study, ECG represented the prolongation of the QT interval, treatment with GA significantly decreased this prolongation. Furthermore, the protective effect of aldose reductase inhibitors in the heart on electrical instability resulting from diabetes has been indicated (40). An experimental study in diabetic rats has demonstrated that GA improved myocardial damage induced by diabetes through inhibiting aldose reductase and increasing anti-oxidant parameters in the heart (14). Effectively, GA decreased QT interval prolongation, a sensitive index of electrical instability, probably via blocking aldose reductase activity and increasing significant anti-oxidant effects. In this study, the QRS complex voltage was decreased in the diabetic rats in comparison with the control group, and GA significantly improved this parameter. The beneficial effects of phenolic and flavonoid agents on the cardiovascular system are exerted by anti-oxidant effects and are also implicated by increasing calcium sensitivity of myofilaments and cardiac contractility (41, 42). In diabetic rats, reduced heart rate is observed, which is associated with the impaired sympathetic nervous system. However, treatment with GA improved this alteration. 

In this study, although the mechanisms involved in the beneficial effect of GA on the heart were not investigated, it suggests that many mechanisms may contribute to improved cardiac electrophysiology and reperfusion-induced arrhythmias by GA treatment, including decreased oxidative stress, inhibition of aldose reductase and calcium channel, reduced cellular communication, increased NO generation and calcium sensitivity of myofilaments. Therefore, more studies are needed on the mechanisms responsible for the protection against cardiac electrophysiology disorders and arrhythmias associated with diabetes by GA. 

## Conclusion

The present study indicated the beneficial effects of GA on cardiac electrophysiology and arrhythmias during reperfusion in diabetes. 
